# Crystal structure of a second polymorph of tetra­kis­(pyridin-2-yl)methane

**DOI:** 10.1107/S1600536814025057

**Published:** 2014-11-21

**Authors:** Kouzou Matsumoto, Masaki Kannami, Akira Fuyuhiro, Masaji Oda

**Affiliations:** a Institute of Natural Sciences, Senshu University, Higashimita 2-1-1, Kawasaki, Kanagawa 214-8580, Japan; b Department of Chemistry, Graduate School of Science, Osaka University, Toyonaka, Osaka 560-0043, Japan

**Keywords:** crystal structure, pyridine, bridging ligands, *S*4 symmetry, polymorph

## Abstract

A second polymorph of the title compound, C_21_H_16_N_4_, is reported. The original polymorph was solved by our group [Matsumoto *et al.* (2003 ▶). *Tetra­hedron Lett.* **44**, 2861–2864] in the monoclinic space group *C*2/*c* and refined to *R* = 0.050. Now the crystal structure of a tetra­gonal polymorph (space group *P*-42_1_ *c*) has been solved and refined to *R* = 0.036. In the crystal, there are no strong inter­molecular inter­actions. Reflecting the high symmetry of the mol­ecular structure, the asymmetric unit is a quarter of the mol­ecule, and the mol­ecule exhibits *S*4 symmetry along the *c* axis in the crystal.

## Related literature   

For a recent review on related bridging ligands, see: Sumby (2011 ▶). For the synthesis of the title compound, see: Matsumoto *et al.* (2003 ▶); Abu-Shanab (2007 ▶). For transition metal complexes of the title compound, see: Matsumoto *et al.* (2004 ▶); Okazawa *et al.* (2004 ▶, 2005 ▶, 2006 ▶); Ishikawa *et al.* (2009 ▶); Hirosawa *et al.* (2012 ▶).
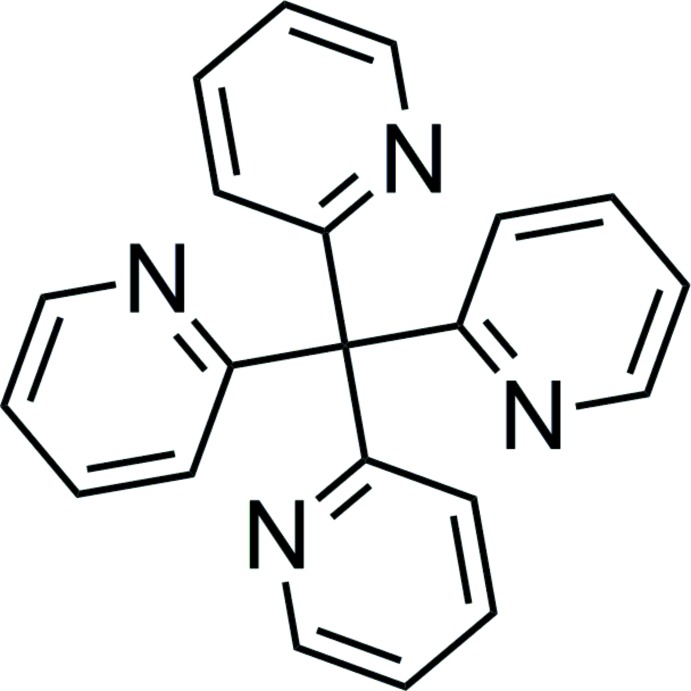


## Experimental   

### Crystal data  

C_21_H_16_N_4_
*M* *_r_* = 324.38
Tetragonal, 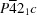

*a* = 10.60 (1) Å
*c* = 7.03 (1) Å
*V* = 790 (2) Å^3^
*Z* = 2
Mo *K*α radiation
μ = 0.08 mm^−1^
*T* = 200 K
0.5 × 0.5 × 0.4 mm

### Data collection  

Rigaku R-AXIS RAPID imaging plate diffractometer
7257 measured reflections
904 independent reflections
858 reflections with *I* > 2σ(*I*)
*R*_int_ = 0.046

### Refinement  

*R*[*F* ^2^ > 2σ(*F* ^2^)] = 0.036
*wR*(*F* ^2^) = 0.090
*S* = 1.07
904 reflections
57 parameters
H-atom parameters constrained
Δρ_max_ = 0.14 e Å^−3^
Δρ_min_ = −0.24 e Å^−3^

### 

Data collection: *PROCESS-AUTO* (Rigaku, 1998 ▶); cell refinement: *PROCESS-AUTO*; data reduction: *PROCESS-AUTO*; program(s) used to solve structure: *SIR2014* (Burla *et al.*, 2012 ▶, 2014 ▶); program(s) used to refine structure: *SHELXL2014* (Sheldrick, 2008 ▶); molecular graphics: *Yadokari-XG 2009* (Wakita, 2001 ▶; Kabuto *et al.*, 2009 ▶) and *ORTEP-3 for Windows* (Farrugia, 2012 ▶); software used to prepare material for publication: *Yadokari-XG 2009* and *publCIF* (Westrip, 2010 ▶).

## Supplementary Material

Crystal structure: contains datablock(s) I. DOI: 10.1107/S1600536814025057/pk2537sup1.cif

Structure factors: contains datablock(s) I. DOI: 10.1107/S1600536814025057/pk2537Isup2.hkl

**Fig. 1.**
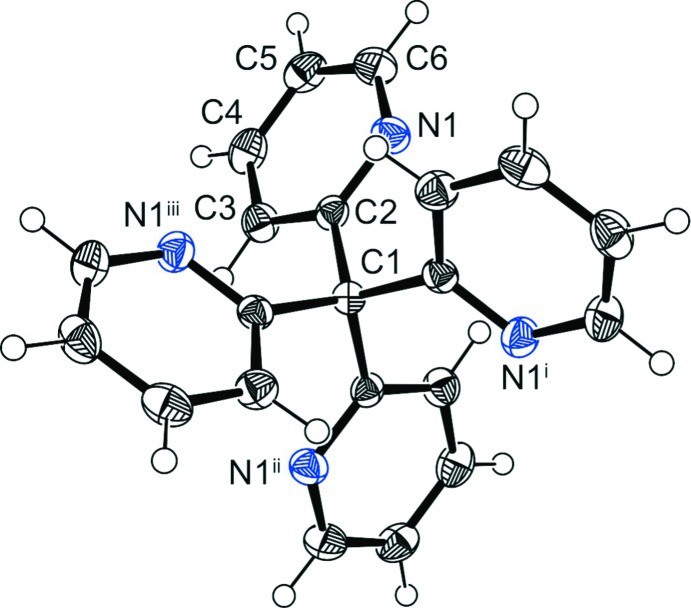
An ellipsoid plot of the title compound (viewed down the c axis). Displacement ellipsoids are drawn at the 50% probability level. Symmetry codes: (i) y, -x, -z + 1; (ii) -x, -y, z; (iii) -y, x, -z + 1.

**Fig. 2.**
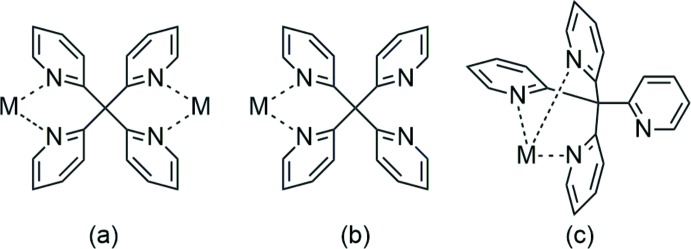
Three kinds of coordination patterns of the title compound. (a) Two-fold bidentate, (b) bidentate, and (c) tripodal. M represents a transition metal ion.

Supporting information file. DOI: 10.1107/S1600536814025057/pk2537Isup3.cml

c y x z x y z y x z . DOI: 10.1107/S1600536814025057/pk2537fig1.tif
An ellipsoid plot of the title compound (viewed down the *c* axis). Displacement ellipsoids are drawn at the 50% probability level. Symmetry codes: (i) *y*, −*x*, −*z* + 1; (ii) −*x*, −*y*, *z*; (iii) −*y*, *x*, −*z* + 1.

a b c M . DOI: 10.1107/S1600536814025057/pk2537fig2.tif
Three kinds of coordination patterns of the title compound. (*a*) Two-fold bidentate, (*b*) bidentate, and (*c*) tripodal. *M* represents a transition metal ion.

CCDC reference: 1034349

Additional supporting information:  crystallographic information; 3D view; checkCIF report

## supplementary crystallographic information

### S1. Comment

Bridging ligands that possess more than one coordination site have attracted considerable attention in relation to multinuclear metallosupramolecular assemblies and coordination polymers (Sumby, 2011). The title compound (Fig. 1) is a typical tetrahedral bridging ligand when it binds to two metal ions in a two-fold bidentate fashion (Fig. 2*a*). The title compound was first synthesized by the reaction of tris(pyridin-2-yl)methyl anion with 2-chloropyridine (Matsumoto *et al.*, 2003). More recently, Abu-Shanab reported that the treatment of 2-picoline with excess of lithium diisopropylamide followed by 2-bromopyridine yielded the title compound as the main product (Abu-Shanab, 2007). The title compound takes on *S*4 symmetry along the *c* axis in the crystal (Fig. 1), reflecting the highly symmetric molecular structure.

The silver complex of the title compound forms a one-dimensional coordination polymer in which the title compound acts as a two-fold bidentate bridging ligand (Matsumoto *et al.*, 2004). This coordination pattern was also observed in a dinuclear Mn(II) complex (Okazawa *et al.*, 2004) and dinuclear Ni(II) complex (Okazawa *et al.*, 2006). On the other hand, the title compound often takes bidentate (Fig. 2*b*) or tripodal coordination patterns (Fig. 2*c*) to bind only one metal ion. The bidentate coordination pattern was observed in the Cu(II) complexes of the title compound (Matsumoto *et al.*, 2004; Okazawa *et al.*, 2005) and tripodal coordination patterns were observed in Cu(II), Fe(II), and Co(II) complexes (Matsumoto *et al.*, 2004; Ishikawa *et al.*, 2009; Hirosawa *et al.*, 2012).

### S2. Experimental

The title compound was synthesized by the reported procedure (Matsumoto *et al.*, 2003). Crystallization from methanol/chloroform (1/1) gave colorless crystals, which were used for X-ray analysis.

### S3. Refinement

Aromatic H atoms were positioned geometrically and allowed to ride on their parent atoms, with C—H = 0.95 Å and *U*_iso_ = 1.2 *U*_eq_(C).

### Crystal data

**Table:**

C_21_H_16_N_4_	Melting point: 259 K
*M**_r_* = 324.38	Mo *K*α radiation, λ = 0.71075 Å
Tetragonal, *P*42_1_*c*	Cell parameters from 6223 reflections
*a* = 10.60 (1) Å	θ = 3.5–27.5°
*c* = 7.03 (1) Å	µ = 0.08 mm^−^^1^
*V* = 790 (2) Å^3^	*T* = 200 K
*Z* = 2	Prism, colourless
*F*(000) = 340	0.5 × 0.5 × 0.4 mm
*D*_x_ = 1.364 Mg m^−^^3^	

### Data collection

**Table:**

Rigaku R-AXIS RAPID imaging plate diffractometer	*R*_int_ = 0.046
Detector resolution: 10.00 pixels mm^-1^	θ_max_ = 27.5°, θ_min_ = 3.5°
ω scans	*h* = −10→13
7257 measured reflections	*k* = −12→13
904 independent reflections	*l* = −9→9
858 reflections with *I* > 2σ(*I*)	

### Refinement

**Table:**

Refinement on *F*^2^	Primary atom site location: structure-invariant direct methods
Least-squares matrix: full	Secondary atom site location: difference Fourier map
*R*[*F*^2^ > 2σ(*F*^2^)] = 0.036	Hydrogen site location: inferred from neighbouring sites
*wR*(*F*^2^) = 0.090	H-atom parameters constrained
*S* = 1.07	*w* = 1/[σ^2^(*F*_o_^2^) + (0.0438*P*)^2^ + 0.1368*P*] where *P* = (*F*_o_^2^ + 2*F*_c_^2^)/3
904 reflections	(Δ/σ)_max_ < 0.001
57 parameters	Δρ_max_ = 0.14 e Å^−^^3^
0 restraints	Δρ_min_ = −0.24 e Å^−^^3^

### Special details

**Table:**

Geometry. All e.s.d.'s (except the e.s.d. in the dihedral angle between two l.s. planes) are estimated using the full covariance matrix. The cell e.s.d.'s are taken into account individually in the estimation of e.s.d.'s in distances, angles and torsion angles; correlations between e.s.d.'s in cell parameters are only used when they are defined by crystal symmetry. An approximate (isotropic) treatment of cell e.s.d.'s is used for estimating e.s.d.'s involving l.s. planes.

### Fractional atomic coordinates and isotropic or equivalent isotropic displacement parameters (Å^2^)

**Table:**

	*x*	*y*	*z*	*U*_iso_*/*U*_eq_	
C1	0.0000	0.0000	0.5000	0.0209 (6)	
C2	−0.02294 (14)	0.11449 (14)	0.3685 (2)	0.0224 (4)	
C3	−0.11972 (16)	0.10889 (16)	0.2328 (3)	0.0290 (4)	
H1	−0.1706	0.0355	0.2218	0.035*	
C4	−0.14016 (17)	0.21120 (17)	0.1155 (3)	0.0340 (4)	
H2	−0.2061	0.2101	0.0241	0.041*	
C5	−0.06207 (18)	0.31595 (17)	0.1342 (3)	0.0337 (4)	
H3	−0.0741	0.3884	0.0567	0.040*	
C6	0.03280 (17)	0.31239 (17)	0.2670 (3)	0.0345 (5)	
H4	0.0873	0.3833	0.2765	0.041*	
N1	0.05294 (13)	0.21395 (13)	0.3845 (2)	0.0283 (4)	

### Atomic displacement parameters (Å^2^)

**Table:**

	*U*^11^	*U*^22^	*U*^33^	*U*^12^	*U*^13^	*U*^23^
C1	0.0205 (9)	0.0205 (9)	0.0216 (14)	0.000	0.000	0.000
C2	0.0216 (7)	0.0233 (7)	0.0222 (7)	0.0020 (5)	0.0033 (6)	0.0006 (7)
C3	0.0279 (8)	0.0315 (8)	0.0275 (8)	−0.0007 (6)	−0.0028 (7)	0.0007 (7)
C4	0.0310 (9)	0.0430 (10)	0.0280 (8)	0.0076 (7)	−0.0026 (8)	0.0038 (8)
C5	0.0393 (9)	0.0316 (9)	0.0302 (9)	0.0077 (7)	0.0046 (8)	0.0093 (8)
C6	0.0382 (10)	0.0268 (9)	0.0386 (10)	−0.0028 (7)	0.0025 (8)	0.0075 (7)
N1	0.0294 (7)	0.0257 (7)	0.0299 (7)	−0.0022 (5)	−0.0014 (6)	0.0039 (6)

### Geometric parameters (Å, º)

**Table:**

C1—C2^i^	1.545 (2)	C3—H1	0.9500
C1—C2^ii^	1.545 (2)	C4—C5	1.391 (3)
C1—C2^iii^	1.545 (2)	C4—H2	0.9500
C1—C2	1.545 (2)	C5—C6	1.373 (3)
C2—N1	1.330 (3)	C5—H3	0.9500
C2—C3	1.402 (3)	C6—N1	1.348 (3)
C3—C4	1.380 (3)	C6—H4	0.9500
			
C2^i^—C1—C2^ii^	111.01 (8)	C2—C3—H1	120.4
C2^i^—C1—C2^iii^	106.43 (16)	C3—C4—C5	118.52 (16)
C2^ii^—C1—C2^iii^	111.01 (8)	C3—C4—H2	120.7
C2^i^—C1—C2	111.01 (8)	C5—C4—H2	120.7
C2^ii^—C1—C2	106.43 (16)	C6—C5—C4	118.55 (15)
C2^iii^—C1—C2	111.01 (8)	C6—C5—H3	120.7
N1—C2—C3	122.22 (15)	C4—C5—H3	120.7
N1—C2—C1	118.44 (13)	N1—C6—C5	123.68 (17)
C3—C2—C1	119.30 (14)	N1—C6—H4	118.2
C4—C3—C2	119.26 (17)	C5—C6—H4	118.2
C4—C3—H1	120.4	C2—N1—C6	117.72 (15)
			
C2^i^—C1—C2—N1	−0.78 (13)	C1—C2—C3—C4	179.97 (14)
C2^ii^—C1—C2—N1	−121.70 (15)	C2—C3—C4—C5	1.1 (3)
C2^iii^—C1—C2—N1	117.39 (18)	C3—C4—C5—C6	0.7 (3)
C2^i^—C1—C2—C3	177.21 (14)	C4—C5—C6—N1	−1.8 (3)
C2^ii^—C1—C2—C3	56.30 (12)	C3—C2—N1—C6	1.2 (2)
C2^iii^—C1—C2—C3	−64.62 (12)	C1—C2—N1—C6	179.09 (13)
N1—C2—C3—C4	−2.1 (2)	C5—C6—N1—C2	0.8 (3)

Symmetry codes: (i) *y*, −*x*, −*z*+1; (ii) −*x*, −*y*, *z*; (iii) −*y*, *x*, −*z*+1.
