# Supplementary figures and images for: Crystal structure of a second polymorph of tetra­kis­(pyridin-2-yl)methane
Source: Acta Crystallogr Sect E Struct Rep Online. 2014 Nov 21;70(Pt 12):o1277–8. doi: 10.1107/S1600536814025057 (PMC4257413; doi:10.1107/S1600536814025057)

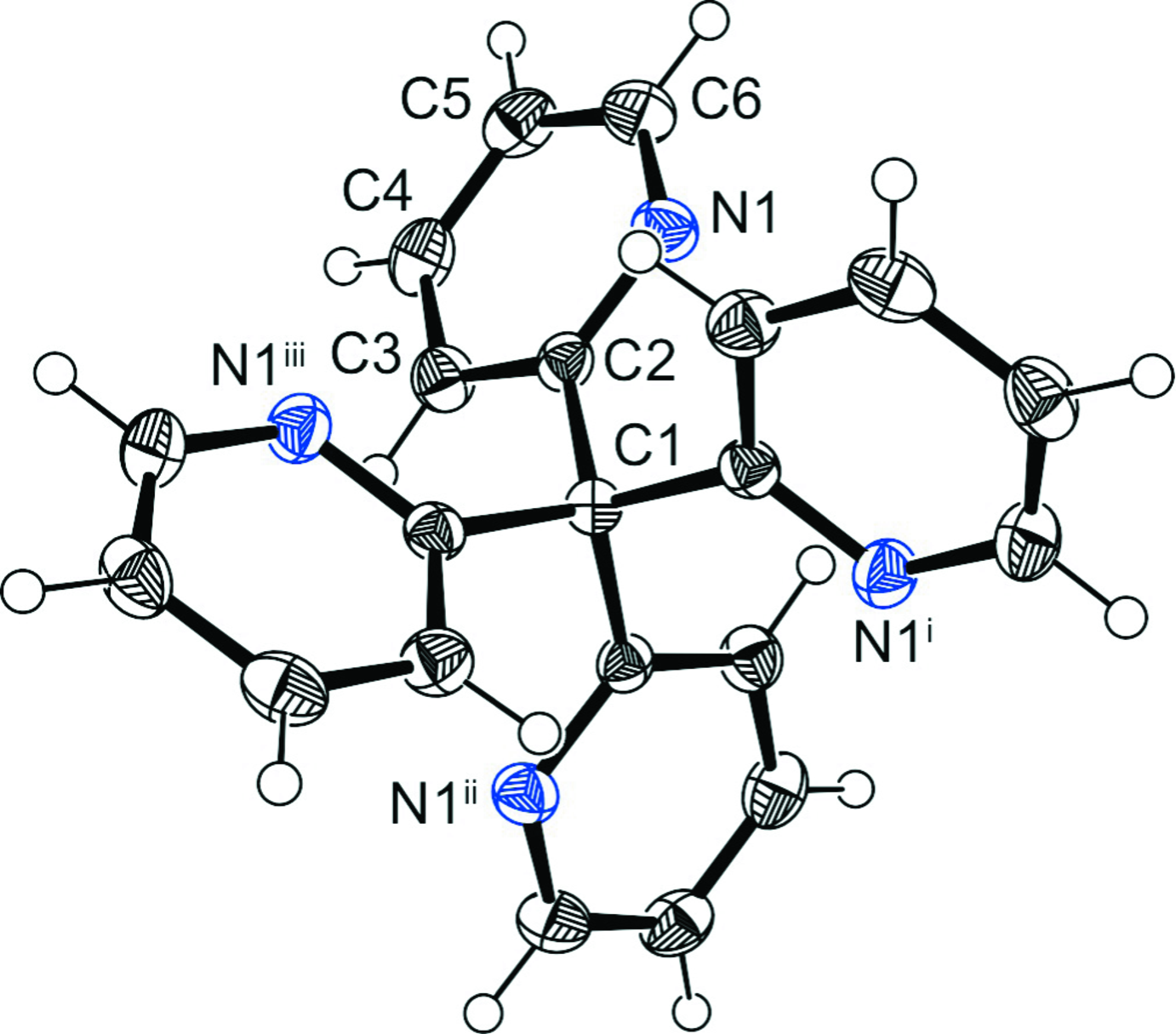

Supplement: Supplementary file 4 [file e-70-o1277-fig1.tif]

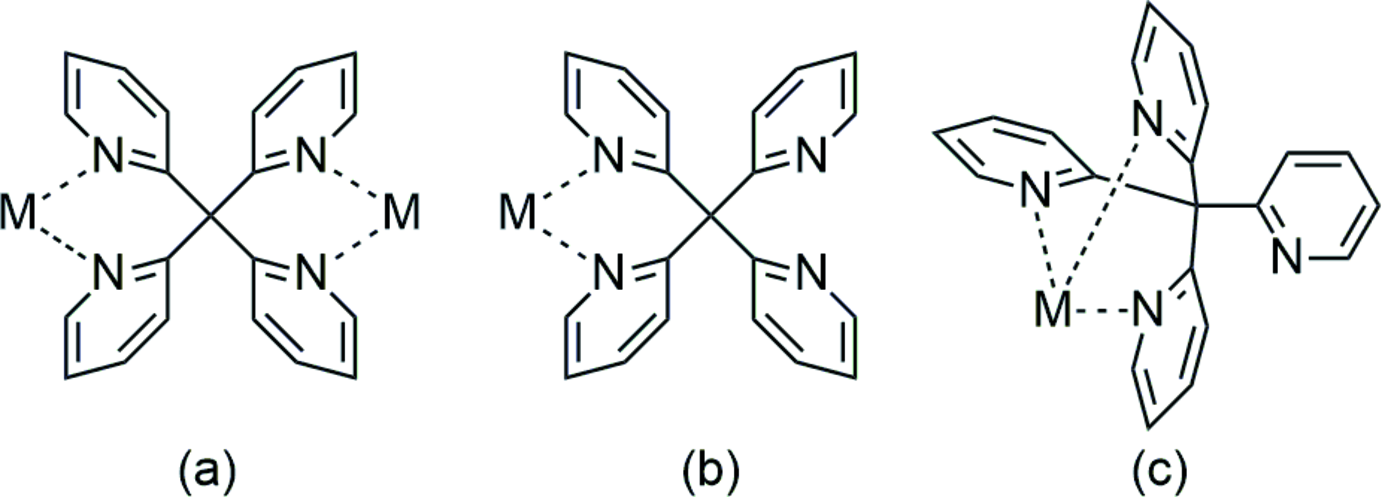

Supplement: Supplementary file 5 [file e-70-o1277-fig2.tif]
